# Farmers Experiencing Potato Seed Degeneration Respond but Do Not Adjust Their Seed Replacement Strategies in Ecuador

**DOI:** 10.1007/s12230-022-09893-0

**Published:** 2022-12-21

**Authors:** Israel Navarrete, Jorge L. Andrade-Piedra, Victoria López, Xuanyu Yue, Jazmín Herrera, Mayra Barzallo, Klever Quimbiulco, Conny J. M. Almekinders, Paul C. Struik

**Affiliations:** 1 International Potato Center, Quito, Ecuador; 2 Centre for Crop Systems Analysis, Wageningen University and Research, Wageningen, The Netherlands; 3 Knowledge, Technology and Innovation, Wageningen University and Research, Wageningen, The Netherlands; 4 CGIAR Research Program on Roots, Tubers and Bananas (RTB), Lima, Perú; 5 International Potato Center, Lima, Perú; 6 Instituto Nacional de Investigaciones Agropecuarias del Ecuador, Quito, Ecuador; 7 Universidad Técnica de Cotopaxi, Latacunga, Ecuador; 8 Ministerio de Agricultura y Ganadería, Latacunga, Ecuador; 9 Universidad de Investigación de Tecnología Experimental Yachay, Urcuquí, Imbabura Ecuador

**Keywords:** Indicators of seed degeneration, Farmers’ knowledge, Farmers’ practices, Local seed systems, Purple top disease

## Abstract

In Ecuador, farmers poorly adopt practices to manage potato seed degeneration. This could be related to the deficient understanding of the farmers’ capacity to experience seed degeneration and respond to it. We contribute to this understanding by answering: How do farmers experience seed degeneration?; What practices do farmers implement when their seed is degenerated?; and Is experiencing degeneration the pivotal factor determining how farmers replace their seed regardless their income? We analysed data collected in Ecuador through farmers’ focus group discussions, farmers’ surveys and interviews, and the Ecuadorian employment status survey. We found that approximately half of the farmers experienced degeneration. Farmers experienced it through low yields, change in seed appearance, crop weakening, and seed physiological problems. When farmers experienced degeneration, they replaced their seed, sought for technical advice, applied more agricultural inputs, or grew other crops. Income was an important trigger for farmers to change their seed replacement practices.

## Introduction

Potato (*Solanum tuberosum* L.) seed degeneration is a threat for potato production in low- and middle-income countries (Thomas-Sharma et al. 2016). Seed degeneration is defined as the process of accumulating pests and pathogens on or in the seed when seed is propagated on-farm over successive cycles. This process leads to a reduction of seed quality and yield (Struik and Wiersema, 1999; Thomas-Sharma et al., 2016). Potato seed degeneration is considered a threat because the use of certified seed and seed replacement rates are low (Thiele 1999; Gildemacher et al. 2009). It is presumably also exacerbated by the low use of improved potato varieties resistant to seed degeneration (Eriksson et al. 2018; McEwan et al. 2021) and the variable adoption of practices to manage seed degeneration (Priegnitz et al. 2019). By implementing on-farm management practices like positive selection, a farmer could increase the yield on average by 12% in comparison with farmers’ seed (Priegnitz et al. 2020). Other practices like seed plot technique could help maintain the health of the seed for multiple cycles of on-farm propagation (Bryan 1983; Kinyua et al. 2015). Despite the potential gains on yield and seed health, farmers are not using these improved practices to manage potato seed degeneration. For example, in Ecuador the use of certified seed is less than 6% (Ministerio de Agricultura y Ganadería 2020), and positive selection, roguing and the seed plot technique are applied by less than 3% of farmers (Navarrete et al. 2022).

There can be different reasons for the low adoption of varieties and practices to manage seed degeneration. These could be: (1) farmers do not experience seed degeneration and therefore they do not consider they need practices to manage it, (2) farmers experience the problem and have their suitable portfolio of practices (no need for new technologies), and (3) farmers experience the problem, but practices are ineffective under their socio-ecological conditions. Here, experience is defined as knowledge and skills of farmers to recognize or identify signs of the process of seed degeneration (adapted from Oxford University Press, 2021). Regarding the mentioned reasons, economic factors such as (on/off) farm income or land availability are important drivers for the decision to adopt or select a practice (i.e., actions that farmers take to manage diseases and pests) (Ojiem et al. 2006; Schreinemachers et al., 2015; Nigussie et al. 2017; Teshome et al. 2019; Islam et al., 2020). In the case of practices that could help manage seed degeneration, there is evidence that farmers’ wealth, investment capacity, and household food insecurity influence the decision process to use certified seed, replace or exchange seed, or use improved varieties (Okello et al. 2016; Tadesse et al. 2017a; Tadesse et al. 2017b). This information highlights the importance of understanding how farmers experience and respond to seed degeneration, and how farmers’ economic conditions influence the type of response(s). A better understanding of this problem could contribute to increasing adoption rates, by bridging the knowledge gaps between farmers and scientists, strengthening farmers’ capacities, and improving communication strategies (van Mele 2000; van Asten et al. 2009; Liebig et al. 2016; Urrea-Hernandez et al. 2016; Duong et al. 2019).

Farmers experience pests and diseases through their ability to observe. They assign folk names to pests and diseases based on their observations such as *Mancha chocolatada* that in Bolivia refers to chocolate spot (*Botrytis fabae*) in beans or *Kuwumba* that in Uganda refers to weevils (Bentley et al. 2009). Farmers also use this ability to evaluate the quality of the seed. In this regard, Peruvian farmers look into the eyes of the potato for soil traces to identify the origin of the seed and hence evaluate the seed quality (Urrea-Hernandez et al. 2016). Unfortunately, when it comes to seed degeneration, the information on how farmers experience this problem is poor. Crissman et al. (1993) found that farmers experience degeneration when the seed does no longer have the capacity to produce well. In the Andean region, farmers refer to this experience as *la semilla está cansada* (translation from Spanish: the seed is tired; Crissman and Uquillas 1989). In Kenya, farmers use the word *Kuzara* that in Swahili means feeble-looking and diseased plants unlikely to provide good yields (Kwame Ogero, personal communication, 2021). However, these descriptions are imprecise because they do not describe whether farmers experience the process of seed degeneration or just the end result of the degenerated seed: low productivity. Unfortunately, the farmers’ description of low productivity is subjective as it can be the consequence of a combination of factors beyond seed degeneration, such as lack of rainfall during the growing season. It is critical to explore other indicators that farmers observe on the degenerated seed.

It is likely that farmers will respond once they experience seed degeneration. One of the most common practices is the replacement of degenerated seed with high-quality or certified seed (Thiele 1999). This replacement could be with seed of the same variety or a different variety (Crissman et al. 1993), with new traits such as disease resistance, etc. Farmers could opt to partially or entirely replace their seed lot or the varieties (Almekinders et al. 1994; McGuire and Sperling 2008). Yet, seed replacement due to seed degeneration seems to be uncommon in the Andes (Crissman and Uquillas 1989; Almekinders et al. 2019) Even though replacing seed is a common farmers’ response, implementing this practice could be challenging for them in certain countries. This is because access to seed could be restricted by the presence of plant diseases or pests or poor availability of seed from different sources (e.g., specialized seed producers or seed potato production programs). For example, the presence of Bacterial wilt (caused by *Ralstonia solanacearum*) impeded farmers’ purchase of seed from seed cooperatives in Ethiopia (Abdurahman et al. 2017). Yet, there is insufficient information to understand how farmers are replacing their degenerated seed or responding to seed degeneration when this practice is not an option. To diversify farmers’ responses to seed degeneration, scientists are advocating the use of an “Integrated Seed Health Strategy” in low- and middle- income countries. This approach combines the use of or replacement with high-quality seed, use of resistant varieties, and use of on-farm management of practices such as positive selection (Thomas-Sharma et al. 2016). Despite the potential these practices offer to the management of degeneration, these will be implemented only if farmers experience seed degeneration and decide to respond to it.

In the tropical highlands of Ecuador, farmers’ potato seed lots are affected by a plethora of seed-borne diseases and pests. Among the main causes of seed degeneration are the Andean weevil (*Premnotrypes vorax*), Potato Virus X (PVX), Potato Virus S (PVS), and black scurf (i.e., hardened sclerotia of *Rhizoctonia solani* attached to the seed) (Fankhauser 2000). However, farmers’ management practices, in this region, could be playing an important role in the rates of accumulation of diseases and pests on the seed (Navarrete et al. 2022). The introduction of the potato purple top disease – a new cause of potato seed degeneration in Ecuador – and of the potato psyllid (*Bactericera cockerelli*) into the country seems to have aggravated seed degeneration, with great impact on potato producers (Caicedo et al. 2015; Castillo et al. 2018; Castillo et al. 2019; Caicedo et al. 2020). A sharp increase in the number of fields infected with purple top disease (hereafter outbreak) was noted by farmers and scientists in 2018–2019 (Navarrete et al. 2021). It is presumed that the impacts of purple top during this outbreak led to a decline in the area cropped to potato in the entire country (Castillo 2020) and the increase in the number of insecticide sprayings (first author’s personal observations). Technical recommendations to manage purple top and the potato psyllid include the use of high-quality seed, monitoring the presence of the psyllid, roguing infected plants, and crop rotation (Cuesta et al. 2021).

Unfortunately, there is hardly any information that systematically describe and analyse how farmers experience and respond to seed degeneration. In this article, we want to fill this knowledge gap by focusing on three research questions: (1) How do farmers experience seed degeneration?; (2) If farmers experience seed degeneration, what practices do farmers implement?; and (3) Is experiencing seed degeneration the pivotal factor determining how farmers select their seed replacement practices regardless their income? By seed replacement practices, we refer to means or channels to obtain new seed that will be used instead of the degenerated seed like seed purchase, selecting seed from potato received as gifts or *ración* (in-kind payment in the form of potatoes received when assisting other farmers during the harvest), and seed exchange. We shed light on these research questions for Ecuador using information collected through focus group discussions, farmers’ surveys, two independent sets of interviews, and the Ecuadorian employment status survey. We expect this article motivates a discussion among scientists, practitioners, and decision-makers about how to increase technology adoption by recognizing farmers’ capacity to experience seed degeneration and respond to this problem depending on their economic conditions.

## Materials and Methods

### Context of the Studies

**Fig. 1:**
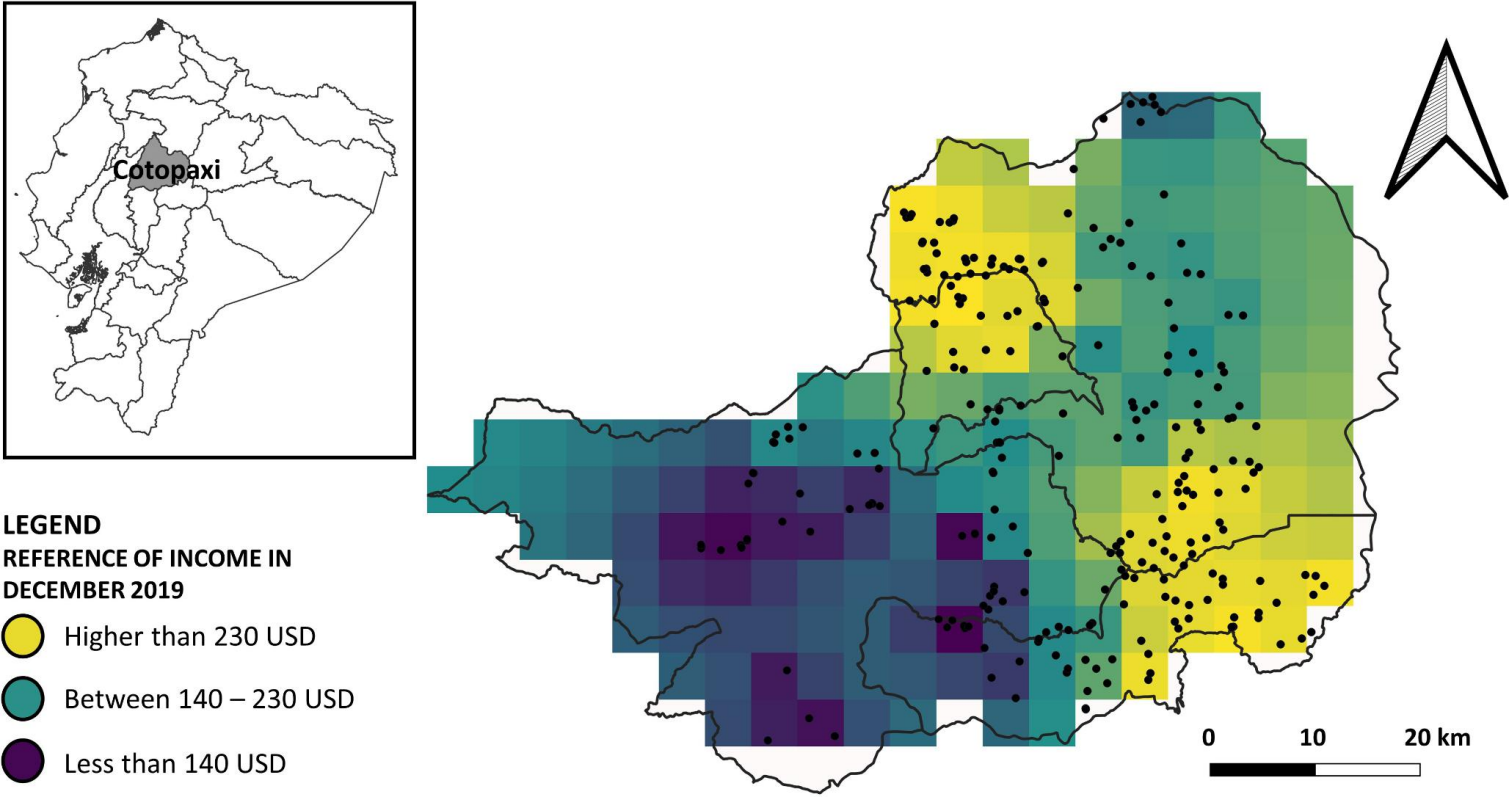
Geographical and economic context of the province of Cotopaxi in Ecuador. On the left, the province of Cotopaxi (grey area) in Ecuador. On the right, main potato-production cantons in the province of Cotopaxi. The grid squares with different colors indicate the variation in income in December 2019 (Instituto Nacional de Estadística y Censos 2019). Dots represent the places where a farmer was surveyed.

We performed this research in the province of Cotopaxi located in the central highlands of Ecuador (Fig. 1). A high proportion of inhabitants of this province are poor (Cabrera et al. 2016); particularly those living in the rural areas (dark areas in Fig. 1). It is estimated that the household income was between 22 and 432 USD during the month of December in 2019 – last report before COVID-19 hit Ecuador – (Instituto Nacional de Estadística y Censos 2019). Farmers produce potato in the intervalley and the highland areas (the western and eastern ranges). The potato production takes place between 2400 and 3800 m above the sea level (Andrade et al. 2002). The entire production accounts for 7.1% of the national production (Sistema de Información Pública Agropecuaria 2020). Even though the potato crop is an important source of income, farmers reach the lowest yields at national level (Ministerio de Agricultura y Ganaderia 2019). Also, the large majority of the farmers save seed for the next season (Ministerio de Agricultura y Ganaderia 2019). For farmers, the main concerns in the production of potato are late blight (*Phytophthora infestans*; reported by 58% farmers), purple top disease (unknown causal agent; reported by 19% farmers), and Andean weevil (*Premnotrypes vorax*; reported by 19% farmers) (Ministerio de Agricultura y Ganaderia 2019).

### Data Collection

*Focus groups to discuss how farmers experience seed degeneration and replace seed*: Four meetings were organized with farmers in the province of Cotopaxi from March to June 2018 (Table 1). These meetings were organized by our project partners: the extension department of the National Agricultural Research Institute, the extension department of the Ministry of Agriculture and Livestock, and the Technical University of Cotopaxi. For this reason, these took place in the areas where our partners are implementing seed interventions. At the beginning of each meeting, farmers were asked to list their potato varieties according to the destination: market or home consumption. Only in one meeting, farmers decided to compare bred and local varieties. In total, farmers listed between 8 and 18 potato varieties. Out of these varieties, they selected the two most representative varieties for further discussion (one bred and local variety; Table 1). In total of the four meetings, farmers were divided into 15 small focus groups (hereafter FGs) each with approximately 7 men or 7 women per group; within groups, gender was not mixed to make participants feel comfortable. A facilitator (of the same gender as the group members) guided in Spanish each FG during the discussion about their seed systems and seed degeneration. During the discussion, we asked farmers: (1) do your seeds degenerate or do you observe your seed ‘to become tired’? and (2) which of the selected varieties - or variety type in the case of the first meeting - degenerate faster? A representative of each FG reported the group’s main points in a plenary session. Afterwards, facilitators met to share the insights obtained in each FG. The results of these meetings contributed to design the questionnaire of a survey.

**Table 1:** Characteristics of meetings with farmers and synthesis of the main results of the focus groups discussions

					Source of degeneration according to farmers (n of groups)				Seed replacement practices (n of groups)^1^			
**Canton**	**Farmers’ groups** **(n)**	**Varieties listed** **(n)**	**Varieties selected for discussion**		**Seed**	**Soil**	**None**		**Ración**	**Gifts**	**Seed exchange**	**Purchase**
Latacunga	4	18	Superchola and Leona Negra		2	2	.		1	3	1	3
Pujilí	4	16	Superchola and Leona Negra		2	2	.		1	2	.	3
Saquisilí	4	8	Bred and local potato varieties		3	.	1		1	.	.	1
Saquisilí	3	15	Superchola and Leona Negra		.	3	.		.	2	.	2
^1^Groups often mentioned more than one mechanism of seed replacement												

*Surveys to understand how farmers experience seed degeneration and replace seed (“Surveys”):* The methodology and the main results of this survey were already reported by Navarrete et al. (2022). Briefly, we surveyed 260 households in the province of Cotopaxi. We selected the place to perform the surveys by overlapping the altitude map of the province of Cotopaxi with a map containing 260 grid squares. We surveyed one household approximately at the centre of each grid square. In each household, we asked farmers: (1) do you experience or have you experienced seed degeneration or that your seed ‘is becoming tired’?; (2) how do you experience seed degeneration or seed ‘being tired’? In addition, we asked if they performed any of the following seed replacement practices: using *ración* (i.e., in-kind payment in the form of potatoes received when assisting other farmers during the harvest) as a seed source, using potatoes received as gifts as seed source, seed exchange, and purchasing seed. There is the possibility that we ignored other seed replacement practices not mentioned during the FGs.

*Telephone interviews and “purple top surveys” to understand farmers’ practices when seed gets degenerated*: We performed two studies to identify practices when farmers experience that their potato seed is degenerated. In the first study, we interviewed 52 farmers from September to October 2020. Due to COVID-19 restrictions, we only interviewed farmers that were able to talk by phone, all being farmers that were part of the Technical University of Cotopaxi’s network. Farmers were interviewed for approximately 10 min. During this interview, we asked farmers in Spanish: (1) do you live in the intervalley or in the highlands?; (2) have you experienced that the seed is degenerated or seed ‘is becoming tired’?; and (3) what practices do you implement when noticing seed degeneration/seed is tired? In the second study (“purple top surveys”), we focused our attention on the purple top disease and two practices that farmers could implement to respond to this problem: crop replacement and increase in insecticide sprayings. For this, we surveyed (face-to-face) 22 farmers in November 2019 after the outbreak of purple top disease in the province of Cotopaxi. These farmers were selected because: (1) they represented smallholder farmers from the potato production cantons of the province, (2) they were interested in training about purple top, and (3) they were actively participating in seed projects led by the University of Cotopaxi and the Ministry of Agriculture and Livestock. We asked farmers: did you observe purple top disease in your fields?; what were your crops during and after the outbreak of purple top disease?; and how many insecticide sprayings did you apply to your potato crops during and after the outbreak?

### Data Analysis

#### To Understand how Farmers Experience Seed Degeneration (FGs and Surveys)

We analysed the data from the FGs in different steps. In the first step, we transcribed the information of each FG into an Excel file. Then, we summarized and compiled the main insights of each meeting. We classified the summaries by the type of varieties discussed in the same Excel file to have a complete raw dataset. Next, we uploaded this Excel file in NVIVO (version 1.3; QSR International) and labeled the description of seed degeneration provided by the farmers. These labels were grouped according to their topics and named “indicators of seed degeneration”. These indicators were identified in the survey’s description of seed degeneration. We counted the number of farmers mentioning each of the indicators of seed degeneration.

*To understand practices implemented when the seed is degenerated (telephone interviews and purple top surveys):* The data of telephone interviews was uploaded into NVIVO (version 1.3; QSR International). Then, we labeled the practices that farmers performed when their seed was degenerated. We grouped together similar topics and identified these groups as: practices adopted by farmers when experiencing seed degeneration. Then, we calculated the frequency of these practices. The data of the purple top surveys was labeled to understand if farmers changed their practices (crop replacement and variation in insecticides sprayings) after the outbreak of the purple top disease. Using R (version 4.0.2), we analysed these data using cross tabulation between farmers who experienced or did not experience their fields infected by purple top. We performed a Pearson’s Chi-Square to evaluate if these changes in practices were associated with farmers’ experiences. For the variation in number of insecticide sprayings, we performed the Pearson’s Chi-Square only on the farmers who continued planting potato after the purple top outbreak. We also reported the number of farmers who did not remember the number of insecticide sprayings during and after the outbreak.

*To understand the influence of farmers’ experience on seed degeneration and income on the seed replacement practices (Surveys):* Before performing this analysis, we downloaded the income data (in US dollars; USD) from the Ecuadorian employment status survey (in Spanish ENEMDU) (Instituto Nacional de Estadística y Censos 2019). We downloaded the data from December of 2019 because December is a representative month for the period in which farmers were surveyed and data of 2018 was not available. Using QGIS (version 3.14), we averaged income information per parish and extracted the data using the geographic positions of each survey. In the case that parish information was not available, we estimated the income based on their location assuming that closer parishes would have a similar income. Then, we performed a logistic regression in R (version 4.0.2) to understand if there is any adjustment on the seed replacement practices due to farmers’ experience on seed degeneration and income (Eq. 1). The following four seed replacement practices were analyzed: selecting seed from ración (Srm_r_), selecting seed from gifts (Srm_g_), seed exchange (Srm_e_), and seed purchase (Srm_p_).

1 $$Sr{m_{r,g,e,p}}\, \sim \,Farmers\,experiencing{\rm{ }}seed{\rm{ }}degeneratio{n_j}\, + \,Estimated\,incom{e_k}\, + \,{\varepsilon _{jk}}$$

**Equation 1.** Logistic regression to understand adjustments of seed replacement practices according to farmers’ experience on seed degeneration and income

## Results

### Farmers’ Observations About Potato Seed Degeneration

The results of our survey showed that 52.7% of farmers had experienced seed degeneration (Table 2). The majority of these farmers (93.4%) described seed degeneration as a seed problem (Table 2). There were few other farmers claiming to experience seed degeneration, but their descriptions were related with the soil getting tired (5.9%) or the effect of drought (0.7%; Table 2). Farmers reported that they experienced seed degeneration through: (1) low yield, (2) change in the physical appearance of the seed, (3) crop weakening (i.e., the crop becomes more susceptible to diseases and requires more agricultural inputs), and (4) seed physiological problems (e.g., the seed is old at the moment of planting) (Table 2). From all the indicators, the one most frequently mentioned was low yield, and it was mentioned by 84.3% of farmers (Table 2). The second most frequent indicator was the change in seed physical appearance (18.1%) (Table 2). The other two remaining indicators were mentioned by less than 10% of the farmers (Table 2).

**Table 2:** Numbers and percentages of farmers experiencing and not experiencing seed degeneration, their descriptions, and indicators used to experience this problem in surveys and telephone interviews

	n	(%)
***Surveys***		
Total number of surveys in 2018	258	100
Farmers not experiencing seed degeneration reported in surveys	122	47.3
Farmers experiencing seed degeneration reported in surveys	136	52.7
** Description of seed degeneration**^**1**^:		
Seed problem	127	93.4
Soil problem	8	5.9
Drought problem	1	0.7
** Indicators of seed degeneration**^**2**^:		
Low yield		84.3
Change in seed physical appearance		18.1
Crop weakening		9.5
Seed physiological problems		1.5
***Telephone interviews***		
Total number of telephone interviews	52	100
Farmers not experiencing seed degeneration in telephone interviews	5	10
Farmers experiencing seed degeneration in telephone interviews	47	90
** Practices**^**3**^:		
Seed replacement		74
Technical advice		13
Application of extra agricultural inputs		11
Crop replacement		2
^1^The percentage is estimated from the number of farmers experiencing seed degeneration in the surveys. ^2^The percentage is estimated from the number of farmers that described seed degeneration as a seed problem in the surveys. ^3^The percentage of farmers implementing practices to manage seed degeneration is estimated from the number of farmers experiencing seed degeneration in the telephone interviews.		

The analysis of our 15 FGs showed contrasting observations (Table 1). Seven out of the 15 FGs mentioned that farmers had experienced degeneration of the seed (Table 1), which they referred to as their seed “was tired”. Farmers associated this problem with yield losses and changes in physical appearance (e.g., change in skin colour) of the seeds. In five out of these seven FGs, farmers reported that improved and native varieties degenerated at similar rates, whereas for the other two FGs, farmers reported that improved varieties were more resistant than native varieties or did not degenerate at all. On the other hand, in seven out of the 15 FGs (Table 1), farmers mentioned that the seed did not degenerate. Instead, they mentioned that the “*soil got tired”* (i.e., local term to describe that the soil becomes a yield-limiting factor) because potato was planted more than once in the same field (farmer description). There was only one FG out of the 15 FGs which mentioned that neither the seed nor the soil degenerated (Table 1).

### Practices when Farmers Experience Their Seed is Degenerated

In our telephone interviews, we found farmers who had not experienced that their seed was degenerated (10%) and farmers who had experienced this problem (90%). We identified that farmers who had not experienced that their seed was degenerated discriminated between high-quality and poor-quality seed. From the farmers who had experienced that their seed was degenerated, we found that they had four practices in response to this problem: (1) seed replacement, (2) seeking technical advice, (3) applying extra agricultural inputs, and (4) crop replacement (Table 2). Seed replacement was the most frequent practice when farmers experienced that their seed was degenerated (74%). A farmer described during the interview that he had sold his entire lot of degenerated seed in the market -and then bought new seed- or used the degenerated seed as animal feed. Seed replacement was followed by seeking for technical advice (13%) (Table 2). Application of agricultural inputs was the third most mentioned practice (Table 2). For farmers, this usually included pesticide sprayings and fertilizer applications. Crop replacement was the least common practice when farmers experienced that their seed was degenerated (Table 2).

When focusing on purple top, we found that 10 farmers experienced that their fields were infected with purple top and 12 who did not experience this problem. Most farmers that experienced that their fields were infected with this disease either replaced their crops or increased the number of insecticide sprayings (part of the application of agricultural inputs) (Table 3). We found that 7 out of the 10 farmers that experienced that their fields were infected with this disease replaced their crops, but there were a few farmers that did not implement this practice despite experiencing the disease (3 out of the 10 farmers; *P* < 0.05) (Table 3). The majority of farmers who did not experience that their fields were infected (10 out of the 12 farmers) continued planting potato in the following season after the outbreak. From the farmers who continued planting potatoes (n = 13 farmers), we found that all increased the use of insecticides after being affected by purple top (Table 3). When farmers’ fields were not affected by purple top, most farmers (7 out of the 10 farmers not affected by this problem) did not intensify insecticide spraying. Only one farmer did it after the outbreak (Table 3). We found an association between the increase in number of insecticide sprayings and farmers experiencing that their fields were infected with purple top (*P* = 0.05) (Table 3). We did not register a decrease in the frequency of insecticide sprayings.

**Table 3:** Practices implemented and options of management when farmers experienced purple top disease in their fields in comparison with farmers who did not experience purple top (n = 22 farmers)

		Farmers experiencing that their fields were:	
**Practice**	**Options of management**	**Affected by purple top**	**Not affected by purple top**
Crop replacement			
	No crop replacement	3	10
	Crop replacement	7	2
*P*		0.04	
Number of insecticide sprayings of farmers who continued planting potatoes after the outbreak of purple top			
	No change in the number of insecticide sprayings	0	7
	Increase in the number of insecticide sprayings	3	1
	Information not available		2
*P*		0.05	

### The Effect of Experiencing Seed Degeneration and Farmers’ Income on Seed Replacement Practices

**Fig. 2:**
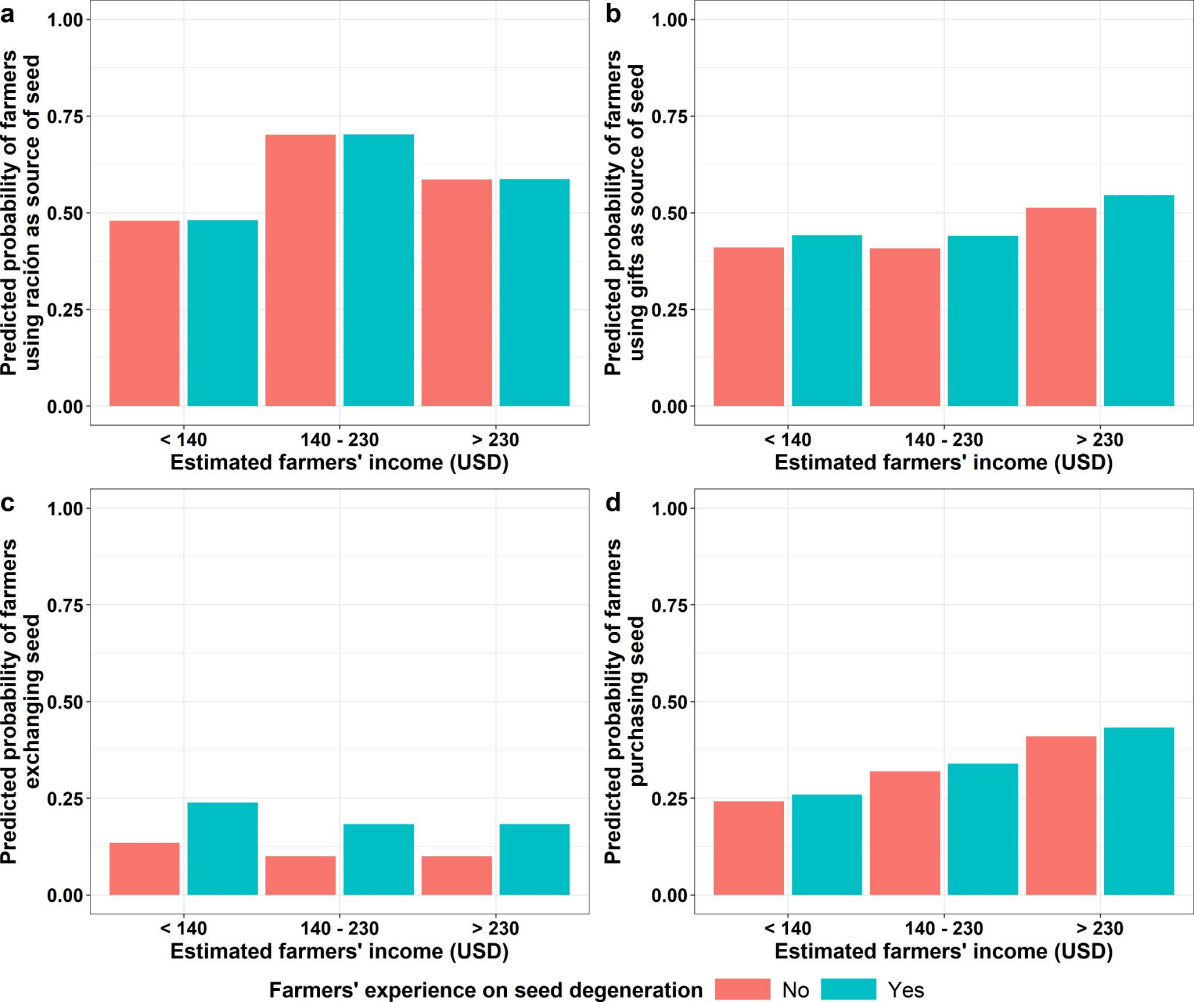
Influence of farmers’ experience on seed degeneration and estimated farmers’ income (USD) on seed replacement practices. (a) Predicted probability of farmers using seed from *ración*. (b) Predicted probability of farmers using seed from potatoes received as gifts. (c) Predicted probability of farmers exchanging seed. (d) Predicted probability of farmers purchasing seed

In the FGs we identified four seed replacement practices: using *ración* as a seed source, using potatoes received as gifts as a seed source, seed exchange, and seed purchase (Table 1). In the surveys, we found the same seed replacement practices. Our surveys’ results showed that from the farmers who received *ración* (n = 130) and gifts (n = 213), we found that 60.8% and 47.4% of them selected seed from these sources, respectively. Moreover, our analysis showed that farmers’ experience on seed degeneration did not influence the probability of farmers using *ración* as a seed source (*P* > 0.05) (Fig. 2a). The estimated farmers’ income did not play a role either (*P* > 0.05) (Fig. 2a). Similarly, the farmers’ experience on seed degeneration and income did not have an influence on the probability of farmers using potatoes received as gifts as a source of seed (*P* > 0.05) (Fig. 2b). Moreover, we found that 15.1% of farmers exchanged seed in the province of Cotopaxi. We found that farmers’ experience on seed degeneration probably affected the decision to exchange seed (*P =* 0.06). Our model estimated that farmers that experienced seed degeneration in general were more likely to exchange seed than farmers with no experience of seed degeneration (Fig. 2c). Farmers’ income was also an insignificant factor determining seed exchange (*P* > 0.05). Overall, in the province of Cotopaxi, 36.0% of the farmers purchased seed when they needed it. Our analysis showed that seed purchase was not affected by the farmers’ experience on seed degeneration (*P* = 0.7) (Fig. 2d). However, it was affected by the farmers’ income (Fig. 2d). Farmers with more than 230 USD of income were significantly more likely to purchase seed (*P* = 0.05) than farmers whose income was lower than 230 USD (Fig. 2d).

## Discussion

Understanding how farmers experience seed degeneration and implement practices to manage it is critical to improve the adoption of improved technologies. We showed that farmers experienced degeneration not only in the seed but also in the soil. Yet, approximately half of the farmers had not experienced this problem at all. Farmers experienced degeneration when the seed did not produce an adequate yield, when the seed tubers changed in physical appearance, when their potato crops weakened, and when the seed had physiological problems. When their seed was degenerated, farmers replaced their seed, sought for technical advice, applied more fertilizers or pesticides, or replaced the crop. For example, it was evident that farmers planted other crops and increased the number of insecticide sprayings after their potato fields were infected by purple top (as hypothesized already by scientists). We showed that the estimated farmers’ income influenced the way how farmers replaced their seed, but we did not find the same for the farmers’ experience on seed degeneration. Scientists and practitioners, in collaboration with farmers, should consider these results to co-design and improve, or adapt seed management practices. The implications of our findings are discussed in the following paragraphs.

### Experiencing Seed Degeneration

Our results showed that 52.7% of farmers had experienced seed degeneration in the province of Cotopaxi (Table 2). They experienced this problem through low yields, change in the physical appearance of the seed, crop weakening, and seed physiological issues (Table 2). These indicators support the descriptions of Thiele (1999) and Urrea-Hernandez et al. (2016), who found that the reduction in productivity and increase in deformed tubers were features that farmers associated with degenerated seed in the Andes. Even though low yields were the most frequent indicator for seed degeneration (Table 2), it is unclear what farmers meant by low yield. Did they mean that the yield obtained in that specific cycle of on-farm propagation was lower than their expectations? Or were farmers experiencing the progressive decline in yield over the successive cycles of on-farm propagation? Further studies to answer these questions could provide important insights about when farmers are motivated to replace their seed.

On the other hand, we identified that farmers experienced degeneration in the soil (Table 2). Farmers in Peru also described similar observations and referred to it as well as *the soil got tired* (Orrego and Andrade 2016; Urrea-Hernandez et al. 2016). This expression, according to farmers participating in the FGs, alluded to the successive planting of potatoes in the same field. Theoretically, under this scenario, there could be an accumulation of soil-borne pathogens such as bacteria, insects, fungi or nematodes. The potato cyst nematodes (*Globodera pallida* and *G. rostochiensis*) and Rhizoctonia (*Rhizoctonia solani*) are examples of pathogens that increase in density every season when potato is continuously planted in the same field (Gilligan et al. 1996; Contina et al. 2020). In the case of Rhizoctonia, higher inoculum densities translate into a larger incidence of degenerated tubers with black scurf (Tsror and Peretz-Alon 2005; Atkinson et al. 2010). Similarly, the cultivation of potato in the same field could contribute to the increase of pests such as the Andean weevil as observed by Rios and Kroschel (2011). This evidence suggests that farmers’ capacity building about seed degeneration should include not only the description and management of seed-borne problems (e.g., viruses), but also the ones in the soil that usually could be spread by the seed.

We found that approximately half of the farmers did not experience seed degeneration (Table 2). This could be explained because farmers cannot observe the symptoms of diseases and pests causing seed degeneration (Nelson et al. 2001) or because some pests and diseases are asymptomatic and their effect in yield is low. For example, PVS is widely spread in the Andes, but this virus causes mild or no symptoms and does not have a significant effect on yield (Santillan et al. 2018). This lack of evident symptoms of diseases and pests causing degeneration could challenge farmers’ observation capacity and might indicate the need to strengthen their capacities on visual diagnostic of pests and diseases that cause seed degeneration (Tafesse et al. 2018; Onditi et al. 2020). Another explanation for the high percentage of farmers not experiencing seed degeneration (not referring to degeneration caused by purple top disease) could be related to the lack of association between variation in yield and level of seed degeneration in farmers’ seed lots (Navarrete et al. 2022). In other words, it might be more difficult for farmers to experience seed degeneration when yield reductions are poorly associated with this problem than when yield reductions are associated with seed degeneration as in the case of the purple top disease (Liefting et al. 2009; Castillo et al. 2018).

### Practices to Manage Seed Degeneration

When farmers experienced that their seed was degenerated, we found that farmers replaced their seed, sought technical advice, applied agricultural inputs (e.g., insecticides), or replaced their potato crop with other crops. From all these practices, we found that seed replacement was the most common practice (Table 2). Our results suggest that farmers did not use as planting material what they identified as degenerated seed. In other words, our results contribute to the hypothesis that farmers will continue using their seeds as long as they obtain the yields that meet their expectations (Crissman et al. 1993; Zeven 1999). Under the Andean context, seed replacement does not necessarily mean that farmers will acquire certified seed; they could also procure apparent good-quality seed from other sources like markets, relatives or neighbours (Almekinders et al. 1994; Thiele 1999). Beyond seed replacement, we identified other practices that can be useful to reduce the rates of seed degeneration. For instance, the application of insecticides could reduce seed degeneration rates caused by Potato Virus Y (PVY), but only when this virus is transmitted by vectors (Hegde et al. 2020). Crop replacement could help to drastically reduce seed degeneration since there is removal of the potato as an inoculum source (Scholthof 2007), and also it could contribute to increase diversity in landscape composition which has an effect on the presence of viruses like PVY (Claflin et al. 2017). Our evidence suggests that farmers are willing to manage seed degeneration, and that they have a portfolio of practices that can be implemented when their seed is degenerated.

Our results also show the flexibility of farmers’ practices portfolio to manage the degenerated seed. We found that seed replacement was the preferred practice when the purple top disease was not the main cause of seed degeneration (Table 3). However, we noticed that when purple top was the cause of seed degeneration, farmers significantly replaced potato with another crop and increased the number of insecticides sprayings (Table 3). There are other practices that farmers are implementing to escape purple top and its potential vector, the potato psyllid, that we did not explore in our studies: abandoning the potato fields infected with this problem and planting in the *páramos* (fragile ecosystem above the tree line) (Navarrete et al. 2021). In any case, our results highlight that the repertoire of practices gives farmers the flexibility to decide what, when and how to manage seed degeneration under their social and ecological context. Similar results were reported by Trutmann et al. (1993) where farmers used this flexibility to increase the efficiency of their practices depending on the agroecological conditions. Yet, it is important to consider that current farmers’ practices might become less efficient in the future due to the uncertain increase of pests and diseases associated with climate change and variability (Perez et al. 2010; Chakraborty and Newton 2011).

### Do Farmers’ Experience on Seed Degeneration and Income Affect Seed Replacement Practices?

We found that there were no differences in using *ración* or potatoes received as gifts as sources of seed when farmers had or had not experienced seed degeneration (Fig. 2a and b). Likewise, we found that seed exchange and purchase were similar among farmers who had experienced seed degeneration and those who had not (Fig. 2c and d). Previous research in the Andes of Ecuador supports our findings indicating that farmers do not primarily replace their seed because of seed degeneration (Crissman and Uquillas 1989). It seems logic that farmers do not replace their seed because of degeneration since this problem is not the main yield-limiting factor under farmers’ context in the Andes of Ecuador (Navarrete et al. 2022). However, there is the possibility that the seed replacement practices do not change because of the experience on seed degeneration, but the source from whom or where farmers obtain the seed. Scientific evidence supports this hypothesis by pointing out that seed quality is one of the criteria for farmers to select their seed sources, regardless the crop (Monares 1988; Bishaw et al. 2010). Yet, we have to acknowledge that these results come from a regression analysis that indicates correlation and not causality. Future research should focus on asking directly whether farmers change their seed replacement practices to avoid seed degeneration.

Our results showed that farmers’ income did not influence all seed replacement practices, except seed purchase. We found a correlation between income and purchasing seed: farmers with higher incomes were more likely to purchase seed than farmers with lower incomes (Fig. 2d). This could be explained by the fact that farmers need to make a decision based on whether they will get a profit in return out of this investment (Bentley et al. 2011). Similar results were identified by Okello et al. (2016) where asset endowment and level of food security were critical variables influencing the purchase of certified seed. Unfortunately, we were not able to break down the effect of on- or off- farm income on the probability of purchasing seed. This information is critical for future seed interventions in the province of Cotopaxi since the majority of the farmers have extremely low incomes (as presented in Fig. 2) and most likely have other priorities than purchasing seed. In the province of Cotopaxi, off-farm income has been identified to play an important role in the economy of smallholder farmers (Caulfield et al. 2019) and an important factor on the management of the farm (Caulfield et al. 2021). On the other hand, our results showed that farmers’ income did not influence the probability of farmers using *ración* or potatoes received as a gift as a source of seed and exchange (Fig. 2). As these practices do not involve monetary transactions, our results confirm that current seed replacement practices are strategies free of cost to maintain seed degeneration at a tolerable level. Overall, our results made evident that seed replacement practices were influenced by farmers’ income rather than by their experience on seed degeneration.

## Conclusion

The results of our paper emphasize the importance of understanding farmers’ experience on seed degeneration and the importance of identifying the available practices to respond to this problem. In our study, we found that approximately half of the farmers had not experienced seed degeneration while the other half had experienced this problem not only in the seed but also in the soil. Therefore, we consider that future seed interventions should not only include practices to manage seed-borne diseases and pests, but also practices that reduce these problems in the soil. Although we identified multiple farmers’ indicators to determine seed degeneration, we found that the low yield was the most popular indicator among farmers. Yet, it remains unknown whether and when low yield could become a motivation for seed replacement. Our results also demonstrated that farmers have a flexible portfolio of practices to manage the different causes of seed degeneration according to their social and ecological conditions. For example, we found that farmers affected by purple top replaced their infected potato fields with another crop and increased the number of insecticides spraying to manage this problem. Besides, our results showed that farmers’ income is the main factor to adjust the seed replacement practices and not the experience on seed degeneration in the Andes of Ecuador. We conclude that it is vital to understand how farmers experience and respond to seed degeneration and their main determinant factors (e.g., income) to identify opportunities to respond when the seed is degenerated.
